# The prevalence of overweight and obesity: a measurement-based analysis of 6–9-year-old school children from Montenegro

**DOI:** 10.3389/fpubh.2023.1194600

**Published:** 2023-05-10

**Authors:** Ivan Vasiljevic, Jovica Petkovic

**Affiliations:** Faculty for Sport and Physical Education, University of Montenegro, Niksic, Montenegro

**Keywords:** anthropometry, monitoring, evaluation, youngsters, Western Balkan

## Abstract

**Introduction:**

This study aimed to conduct a measurement-based analysis of overweight and obesity prevalence among 6–9-year-old children in Montenegro.

**Methods:**

The population of this cross-sectional study included 1993 (1059 boys and 934 girls) primary-school children. The sample of anthropometric variables includes body height, body weight and body mass index, as well as nutrition status that were presented based on BMI standardized categories: underweight, normal weight, overweight, and obesity. The descriptive statistics described the means for each variable, while post hoc tests and ANOVA were applied to explore differences between the proposed means.

**Results:**

The results indicate the overweight (including obesity) prevalence was 28% (15% of overweight and 13% of obese children), while boys have a higher overweight prevalence rate than girls. In addition, the tendency of higher prevalence rates to differ by age in both sexes is observed. This study also confirmed that overweight and obesity are affected by geographical regions but not by the level of urbanization in Montenegro.

**Discussion:**

The innovation of this study is reflected in the fact that the overweight and obesity prevalence rates among 6–9-year-old children are within an acceptable range in Montenegro, since it does not deviate from the European average, but further interventions and continuous monitoring are necessary, due to the specific nature of this issue.

## Introduction

It is widely known that overweight and obesity in children and adolescents are increasing dramatically worldwide (1, 2). This problem was not noticeable during the previous century, but the focus was on underweight; however, there was an expansion of overweight and obesity at the end of the twentieth and the beginning of the twenty-first century (3, 4). The growing trend of overweight and obesity is persistent, dominantly in developed and developing countries, even reaching epidemiological proportions in recent years (2). By changing lifestyles and everyday habits (primarily better personal diet and absence of hard physical work), people have solved the age-old problem of being underweight; however, the time of prosperity did not last long. People went to the other extreme, and a new issue appeared soon: overweight and obesity. The number of countries that have been spared by this issue is low, and overweight and obesity have dramatically threatened the health status of most societies worldwide in the previous two to three decades, with a significant growth trend from year to year. Many studies indicate that the issue exists in all populations (5), from children to the older adult. Nevertheless, obesity in children is the most important issue, first, because it threatens the health status of the youngest population but also because it represents the main risk factor for obesity in adults, so it is necessary to stop this pressing problem at an early stage (6).

Solving overweight and obesity in children is not an easy task, especially because there are a large number of different factors that influence weight gain (7). Also, there is a certain population heterogeneity, i.e., different predispositions in people when it comes to the individual tendency toward this issue, so the level of overweight differs greatly by gender, age, ethnicity, geographical region where people live, as well as by socio-economic characteristics of a certain population (8, 9). Therefore, it is evident that this problem is highly complex and that everything mentioned above indicates that the solution to this global issue is becoming more complicated year by year. At the same time, national protocols for monitoring overweight and obesity around the world are being established, as well as regional and continental ones and even intercontinental initiatives are being launched, which aim to harmonize national monitoring systems and establish unique and comparable protocols in a wider geographical area, such as are SLOfit, FitBack Europe, the WHO European Childhood Obesity Surveillance Initiative (COSI), the Global Observatory for Physical Activity (GoPA), the Global Matrix—Active Healthy Kids Global Alliance, among others (10–15).

When discussing Montenegro, practitioners and researchers from this field actively take part in the international initiatives listed in the previous paragraph and several local initiatives. The employees of the Institute of Public Health and the Faculty of Sports and Physical Education of the University of Montenegro lead in achieving these goals. However, although there have been several attempts, a national initiative and a sustainable national protocol that would continuously, year after year, monitor the entire Montenegrin population and thus create a unique database of high-quality data have not been established yet. As part of the national initiative and, with the help of a sustainable national protocol, a cross-sectional analysis would be carried out, and the trend of changes would be monitored, all to give adequate recommendations for making successful decisions in the process of mitigating the general negative trends in the prevalence of overweight and obesity in Montenegro (16). Therefore, although there is no unique system for monitoring and evaluating the entire Montenegrin population in the manner described (17), certain activities do exist (18), which are based on individual researches with representative samples. Later, the collected data is combined to create a representative database at the national level, which ultimately helps to create the most objective cross-section of overweight and obesity prevalence, as well as to monitor the level of overweight differs by gender, age, ethnicity, geographical region where people live and socio-economic characteristics that is neglected in Montenegro, in comparison to neighboring and European countries (3).

This study aimed to conduct a measurement-based national analysis of overweight and obesity prevalence among 6–9-year-old children from Montenegro to confirm the rising trends in children's overweight and obesity as well as expected differences by geographical regions and level of urbanization.

## Methods

**Figure 1:**
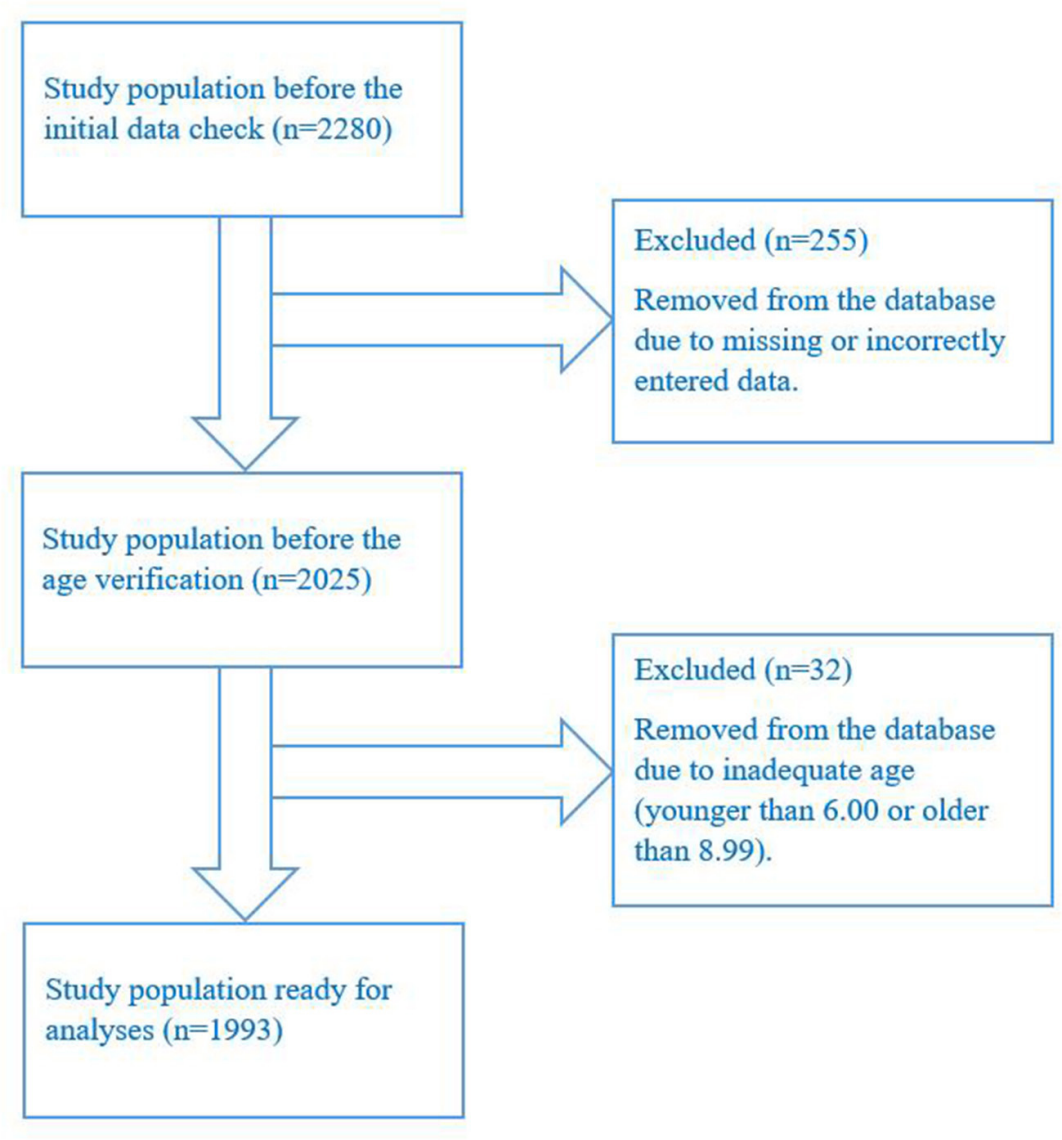
The sampling process.

The population of this cross-sectional study included 1993 (1,059 boys and 934 girls) first- to third-grade primary-school children (average age: 7.96 ± 0.69 years; age range: 6.00–8.99 years) who agreed to participate in the study (parents' informed consent has been obtained on a voluntary basis). The sampling process was performed in line with the COSI Protocol (19) and is shown in Figure 1. Furthermore, cluster sampling is used to divide the study population into multiple groups (clusters), and it is stratified by gender, geographical region, and type of settlement. The study population were assessed for measurement in three Montenegrin regions (southern, central, and northern) and 21 municipalities (out of 25). It was measured from November 2018 to May 2019. Data were collected in line with the Childhood Obesity Surveillance Initiative (COSI) conducted by the Faculty for Sport and Physical Education at the University of Montenegro by trained field staff who performed anthropometric measurements at participating schools.

The principal investigator developed a database for data entry, selected and trained field examiners for data collection, and conducted data quality control (double-checked) in line with the COSI Protocol (19). The sample of variables includes body height and body weight, which were measured to the nearest 0.1 kg and 0.1 cm, respectively, using a scale and a stadiometer (Seca, Hamburg, Germany), while body mass index (BMI) was calculated as body mass (kg) divided by squared stature (m^2^) (20). Nutrition status was presented based on BMI standardized categories: underweight, normal weight, overweight, and obesity (21). In addition to these variables, trained field examiners have also collected the date of birth, date of measurement, gender, as well as school grade, city of residence, geographical region, and type of settlement. Ethical approval was unnecessary as this activity was introduced as voluntary school monitoring within PE classes.

The descriptive statistics were expressed as a mean and standard deviation for each variable, as well as its range, while *post-hoc* tests and ANOVA were employed to explore differences between the proposed means. The data were analyzed statistically using the Statistical Package for the Social Sciences: IBM SPSS Statistics v.20.0.1.0 (IBM, Chicago, IL, USA). Statistical significance was set at *p* < 0.05.

## Results

Analysis of study population characteristics is presented in Table 1. First, the descriptive characteristics of school children are described [i.e., the mean of age, body height (BH), body weight (BH), and body mass index (BMI)] as well as the frequency of respondents in relation to gender, age, geographical region and type of settlement where they live. It is noteworthy that this investigation included 2,280 children from primary schools in 21 municipalities from Montenegro, which indicates a well-distributed sample, since only four municipalities were not included in this study. After the initial data check, 255 respondents were removed from the database due to missing or incorrectly entered data, while 32 children were excluded from further analysis because they were not of adequate age (i.e., they were younger than 6.00 or older than 8.99 years). In the end, the final database contained 1993 children who were included in the analysis.

**Table 1:** Characteristics of sample population (*n* = 1,993).

**Variable**		
Age (years)^a^	7.96 ± 0.69	
Body height (centimeters)^a^	133.05 ± 7.29	
Body weight (kilograms)^a^	30.26 ± 6.49	
Body mass index (kg/m^2^)^a^	16.97 ± 2.66	
**Gender (number, %)** ^b^		
Girls	934 (46.9)	
Boys	1,059 (53.1)	
**Age group (number, %)** ^b^		
6.00–6.99 years	148 (7.4)	[♂92, ♀56]
7.00–7.99 years	742 (37.3)	[♂370, ♀372]
8.00–8.99 years	1,103 (55.3)	[♂597, ♀506]
**Geographical region (number, %)** ^b^ ^,^ ^c^		
Southern	366 (18.4)	[♂202, ♀164]
Central	1,142 (57.3)	[♂612, ♀530]
Northern	485 (24.3)	[♂245, ♀240]
**Type of settlement (number, %)** ^b^ ^,^ ^d^		
Urban	1,661 (83.3)	[♂879, ♀782]
Rural	332 (16.7)	[♂180, ♀152]

a Values are presented as Mean ± SD.

b Values are presented as frequencies (percent).

c Criteria used in categorization of geographical region were administrative classification.

d Criteria used in categorization of the type of settlement were administrative classification and population size, with rural settlement including villages and small towns having 10,000 or fewer inhabitants, and urban having more than 10,000 inhabitants (22).

♂, boys; ♀, girls.

The anthropometric characteristics of the study population are shown in Table 2. The BH, BW, and BMI of the sample population in this study are descriptively depicted, but the mean and standard deviation of the abovementioned variables for all three age groups are also shown, as well as the range values. Boys were taller, heavier and had a higher BMI, both in the total sample and by individual age groups.

**Table 2:** Body height, body weight, and body mass index for individual age and gender groups.

	**Body height (centimeters)**	**Body weight (kilograms)**	**Body mass index (kg/m^2^)**
**6.00–6.99 yrs**.			
Girls	122.27 ± 5.05 (21.6)	23.67 ± 4.82 (22.5)	15.73 ± 2.20 (10.4)
Boys	123.59 ± 5.27 (28.0)	24.55 ± 3.66 (22.8)	16.02 ± 1.72 (10.8)
**7.00–7.99 yrs**.			
Girls	130.74 ± 6.71 (38.2)	28.51 ± 5.87 (29.6)	16.58 ± 2.57 (13.6)
Boys	131.70 ± 6.61 (35.9)	29.60 ± 6.01 (31.4)	16.98 ± 2.63 (13.8)
**8.00–8.99 yrs**.			
Girls	135.16 ± 6.00 (36.5)	31.38 ± 6.23 (34.3)	17.09 ± 2.72 (13.7)
Boys	136.00 ± 6.61 (41.2)	32.32 ± 6.57 (35.6)	17.38 ± 2.75 (14.0)
**Total**			
Girls	132.62 ± 7.09 (42.9)	29.77 ± 6.35 (34.7)	16.81 ± 2.65 (14.1)
Boys	133.42 ± 7.44 (42.2)	30.69 ± 6.58 (37.3)	17.12 ± 2.66 (14.0)

Values are presented as Mean ± SD (Range).

**Figure 2:**
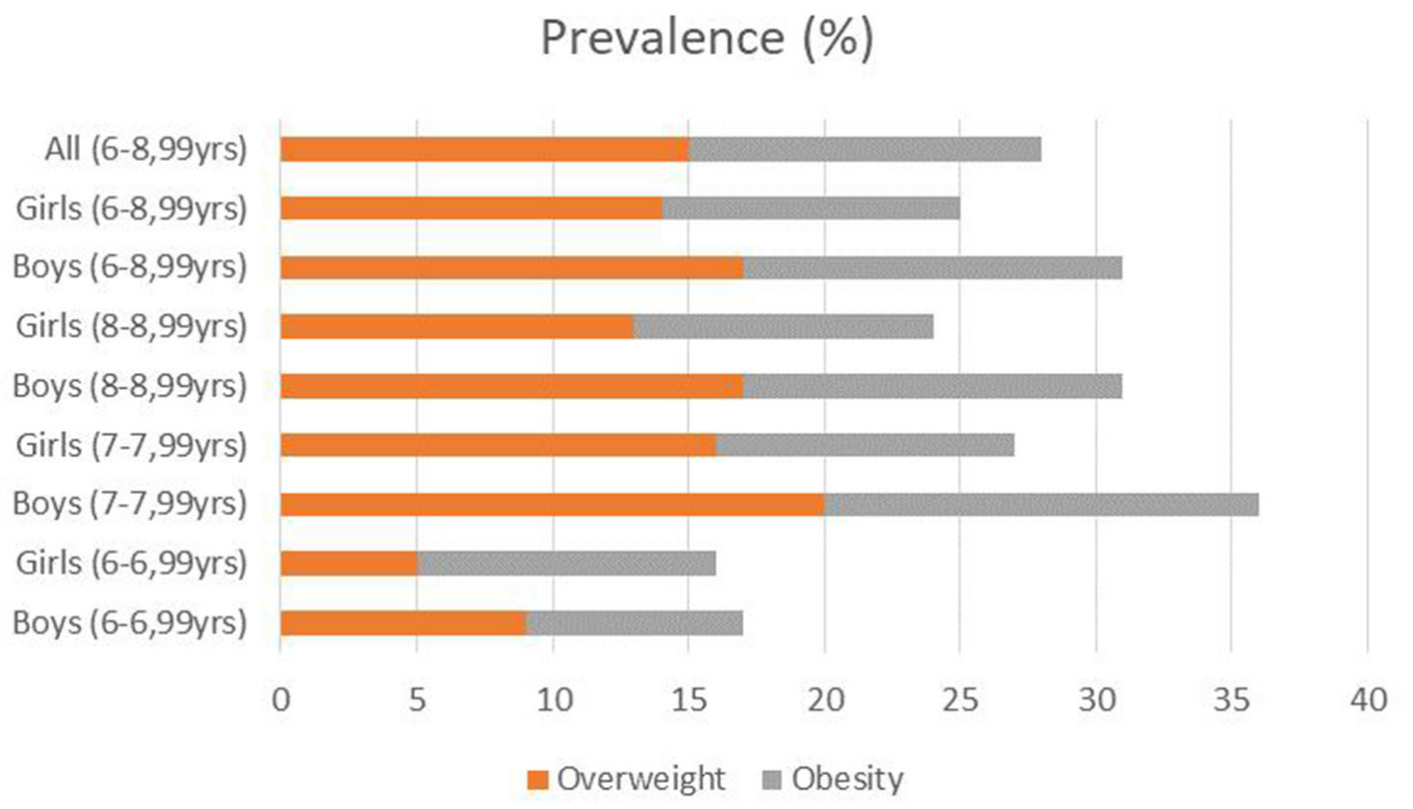
Overweight and obesity prevalence rates among 6–9-year-old children from Montenegro.

Interestingly, significant differences were found in overweight prevalence between boys and girls in equivalent age groups (*p* = 0.042), whereby boys were more likely to be overweight. Overweight and obesity prevalence rates are shown in Figure 2.

This study aimed to determine the influence of geographic region and type of settlement on overweight and obesity of school children. Primarily, it was found that the geographical region significantly affects the overweight prevalence rate among 6–9-year-old children from Montenegro (*p* = 0.01), whereby boys and girls from the northern region were more likely to be overweight (Table 3). In contrast, the level of urbanization did not show any influence on overweight prevalence rates among the study population (*p* = 0.83).

**Table 3:** Overweight rates (obesity included) by geographical region and type of the settlement.

	***n***	**Overweight prevalence^a^**	***p***
**Geographical region**			
Southern	366	30%	
Central	1,142	28%	
Northern	485	27%	0.01^b^
**Type of settlement**			
Urban	1,661	29%	
Rural	332	24%	0.83^c^

a ≥85th percentile.

b The significant difference in overweight rates was found between geographical regions, with *post-hoc* difference reported for children from southern vs. central region (p = 0.04) and southern vs. northern region (p = 0.00).

c The significant difference in overweight prevalence rates was not found among children from different levels of urbanization.

Significant differences in obesity prevalence rates were also found between boys and girls in equivalent age groups (*p* = 0.04), also in favor of girls. The influence of the geographic region and type of settlement on obesity is shown in Table 4. It was also determined that the geographic region significantly affects the obesity prevalence rate among 6–9-year-old children from Montenegro (*p* = 0.01), while the level of urbanization also did not show any influence on the obesity prevalence rates among the study population (*p* = 0.81).

**Table 4:** Obesity rates by geographical region and type of the settlement.

	***n***	**Obesity prevalence^a^**	***p***
**Geographical region**			
Southern	366	14%	
Central	1,142	12%	
Northern	485	14%	0.01^b^
**Type of settlement**			
Urban	1,661	13%	
Rural	332	12%	0.81^c^

a ≥95th percentile.

b The significant difference in obesity rates was found between geographical regions, with *post-hoc* difference reported for school children from southern vs. central region (p = 0.00) and southern vs. northern region (p = 0.00).

c The significant difference in obesity prevalence rates was not found among school children from different levels of urbanization.

## Discussion

Although there is no unique national system for monitoring overweight and obesity among children and adolescents in Montenegro, as has been pointed out several times, individual studies have been carried out occasionally. Furthermore, local institutions were primarily involved in international consortia but also implemented their own national projects, all to solve this contemporary global problem. Therefore, it cannot be stated that this is the first study that assessed the levels of overweight and obesity of this population in Montenegro because the data collected thus far were part of global studies. However, this is one of the pioneer studies at the national level that, from the beginning to the end, applied a standardized European protocol (COSI) and took into account exclusively school children aged 6–9 years.

The results of this study indicate that the overall prevalence of overweight (including obesity) was 28% (15% of overweight and 13% of obese children). These results are similar to those from previous studies that included the Montenegrin population, but a noticeable increase in overall overweight, especially obesity, was observed (18, 23, 24). Compared to other countries (25), it seems that Montenegro does not deviate significantly from the average overweight (including obesity) prevalence rate in the European region, which is also 28%; however, a wide range was observed between individual countries (from 5 to 43%). It is interesting to highlight that the highest rates were found in the Mediterranean countries, such as Spain, Italy, Greece, Malta, and Cyprus (between 38 and 43%), while the lowest rates were found in the countries located in Central Asia, such as Kyrgyzstan, Tajikistan, and Turkmenistan (from 5 to 11%). According to the same report, a large range between individual countries was also found in obesity (from 1 to 22%), which is also dominant, again in Mediterranean countries, and least pronounced in countries located in Central Asia.

Although a slightly higher overweight prevalence rate was observed in boys compared to girls in most European countries, the situation is different in school children aged 6–9 years from Montenegro. Specifically, it has been observed that the boys have a significantly higher overweight prevalence rate. Namely, the overweight (including obesity) prevalence rate was 31% in boys (17% overweight and 14% obese school children), while in girls the rate was 25% (14% overweight and 11% obese school children). It is interesting to indicate that a lower prevalence rate in girls was observed only in France, Portugal, and Slovakia, while in most other European countries, the rate was almost uniform (25). Nevertheless, the situation in Montenegro did not deviate absolutely from the European profile, since, according to the same report, a significant difference between boys and girls was also found in certain countries of the European Union, such as Estonia, Lithuania and Croatia.

In contrast, the gender difference was not the only factor that affects the overweight (including obesity) prevalence rate. A tendency for higher prevalence rates in relation to the age of both sexes is observed in this study. However, the mentioned difference is much more noticeable between the school children who have just started attending school (the first grade) and the older school children who have longer school tenures (the second and third grades). The prevalence rates were significantly lower in the younger population, but drastic differences were not observed in second-grade children and onward. A similar situation was observed in all European Union member states where the data were collected for the purposes of the global report (25); however, the differences were not as striking as in the Montenegrin case. The answer could be found in the fact that children's lifestyle changes significantly when they start attending school; in this way, they move significantly away from natural and traditional lifestyles and adapt to modern lifestyles, which fit into the typical lifestyle of developed and developing countries.

It was assumed that the lifestyle in Montenegro should depend significantly on the geographical region in which people live, as well as on the level of urbanization. Therefore, the hypotheses that were tested in this study were set. In support of this claim, it is important to mention that it is widely recognized that several factors influence the increase in the prevalence of overweight and obesity in childhood, such as the level of physical activity, diet, genetics, socioeconomic status and climate (26). Montenegro is very diverse in this matter, and variations of individual factors could be observed in individual geographical regions and significantly affect the overweight and obesity prevalence rate. Variations between geographical regions were also observed in Italy (27) and Sweden (28), as well as in Malta (29), which is also a small Mediterranean country; so, it is not unusual the similar prevalence rates were determined in this study. Specifically, it is observed in this study that the overall overweight prevalence rates were significantly higher in children living in the southern region (30%) compared to the central (28%) and northern region (27%), while there was no significant difference between children from the central and northern region. Side by side, the differences were also observed in obesity prevalence rates, but significant differences were just found between the central region (12%) and the southern (14%) and northern regions (14%), between which there were no differences. However, care should be taken in drawing conclusions because the mentioned differences may be caused by social inequalities not included in this study's analyses.

In contrast, although it was assumed that it would affect the prevalence rate, no differences were found between populations living in different types of settlements, as is the case in Serbia, where it was found that the risk of being overweight and obesity are strongly associated with a lower level of urbanization (3). A similar situation as in Serbia was observed in Norway (30) and Sweden (31), as well as in Iceland (32), while the risk of being overweight is strongly associated with the urban environment in Turkey and Portugal [cited in Djordjic et al. (3)].

## Conclusion

In conclusion, it should be noted that the overweight and obesity prevalence rate among 6–9-year-old children is within an acceptable range in Montenegro, since it is on the European average. However, this does not mean the situation is good enough and that solving this global problem should be neglected in the coming period. Therefore, it is necessary to undertake certain activities that should be reflected in the design of a sustainable strategy at the national level, which, with the help of decision-makers, would lead (1) to the establishment of a national protocol for the permanent monitoring and evaluation of overweight and obesity prevalence in Montenegro, (2) to apply the adequate interventions that would lead to reductions in the rates of the prevalence of overweight and obesity among Montenegrin school children and bring them as close as possible to the average prevalence rate similar to countries from Central Asia, and (3) to initiate more significant investments in scientific research in the field of health-enhancing physical activity, as decision-makers would receive updated recommendations prepared in accordance with the latest knowledge reached from contemporary scientific research.

However, like any other study, this one is not without certain limitations. The limitation of this study is reflected, above all, in the fact that many cases of overweight and obesity in men and women in Europe can be attributed to inequalities in socio-economic status, whereby obesity and overweight in children are related to the socio-economic status of parents, which this study did not take into account. For this reason, it is obvious there is huge space for upcoming studies that would include the above-mentioned factors in their hypotheses, as well as many other variables related to the use of technology, and, more precisely, answer the research questions this study sets. An additional limitation is related to the fact that a significant number of studies criticized the protocol used in this study and emphasized its shortcomings, especially in the Western Balkans region, where school-age children deviate significantly from their peers from the WHO core reference sample [male: Croatian (5.9 cm), Montenegrin (5.7 cm), Serbian (4.5 cm), Slovenian (4.3 cm), and Macedonian (2.5 cm); female: Croatian (5.4 cm), Montenegrin (4.8 cm), Serbian and Slovenian (4.4 cm), and Macedonian (2.4 cm)]. Therefore, the recommendation for future research is based on the fact that this protocol for assessing the rate of overweight and obesity prevalence among Western Balkan school-age children should be used with caution or some more reliable protocol might be applied such as underwater weighing, measurements with calipers, waist-to-hip ratio measurements, and dual-energy X-ray absorptiometry. Perhaps, machine learning applied to the prediction of obesity and overweight will be one of the potential solutions that might provide solutions in this area in the future.

## Data availability statement

The raw data supporting the conclusions of this article will be made available by the authors, without undue reservation.

## Ethics statement

Ethical review and approval was not required for the study on human participants in accordance with the local legislation and institutional requirements. Written informed consent to participate in this study was provided by the participants' legal guardian/next of kin.

## Author contributions

IV wrote the manuscript, collected the data, performed analyses, and revised the manuscript. JP wrote the manuscript, reviewed previous studies, discussed the results, and revised the manuscript. All authors contributed to the article and approved the submitted version.
